# Occupational exposure to heavy metals: A review of health outcomes and safety interventions

**DOI:** 10.6026/973206300220547

**Published:** 2026-01-31

**Authors:** Meghna Dipakkumar Chaudhary, Rajvi D Chaudhary, Kshitij Sale, Chaitanya Chaudhary, Pranav Manek, Nirav Parmar, Miral Mehta, Dhaval Niranjan Mehta

**Affiliations:** 1 Department of Environmental Health & Safety, Amneal Pharmaceuticals, USA; 2 Health Informatics, Rutgers University, USA; 3 Alumni, Information Technology, Rutgers University, USA; 4 Department of Artificial Intelligence & Machine Learning, Rutgers University, USA; 5 Department of Global and Population Health, Henry M Goldman School of Dental Medicine, Boston University, USA; 6 Department of Conservative Dentistry and Endodontics, Faculty of Dental Science, Dharmsinh Desai University, Nadiad, Gujarat, India; 7 Department of Pediatric and Preventive Dentistry, Karnavati School of Dentistry, Uvarsad, Gandhinagar, Gujarat, India; 8 Department of Oral Medicine and Radiology, Narsinhbhai Patel Dental College and Hospital, Sankalchand Patel University, Visnagar, Gujarat, India

**Keywords:** Occupational health, heavy metals, lead, mercury, cadmium, neurotoxicity, bio-monitoring, safety interventions

## Abstract

Occupational exposure to heavy metals such as lead, mercury, cadmium and arsenic remains a major global health problem, causing significant morbidity despite decades of regulation. Therefore, it is interest to narrative review data on the pathophysiology, clinical effects and diagnostic biomarkers associated with these exposures. Oxidative stress, enzyme inhibition and ionic mimicry explains the widespread neurotoxic, nephrotoxic, cardiovascular and carcinogenic outcomes. Preventive strategies, including engineering controls and rigorous biomonitoring, are evaluated alongside therapeutic options such as chelation. Thus, protecting worker health requires prioritizing primary prevention and strengthening protocols to reduce exposure at its source.

## Background:

The industrial revolution and subsequent technological advancements have profoundly transformed modern society, but not without significant public health consequences. Among the most enduring of these is the health risks associated with occupational exposure to heavy metals [1]. Heavy metals are a group of naturally occurring elements characterized by high density and atomic weight, many of which are systemically toxic even at low concentrations. Metals such as lead (Pb), mercury (Hg), cadmium (Cd) and arsenic (As) are indispensable in numerous industrial processes, including mining, smelting, battery manufacturing, electroplating, welding and agriculture [2]. Consequently, millions of workers worldwide are at risk of exposure through inhalation of dust and fumes, dermal absorption, or ingestion. The significance of this topic is underscored by the persistent and cumulative nature of heavy metal toxicity. Unlike many organic toxins, metals are not readily metabolized and can accumulate in biological tissues over a lifetime, leading to a delayed onset of chronic, debilitating diseases [3]. This bioaccumulation complicates the establishment of direct causal links and safe exposure limits, presenting an ongoing challenge for occupational health practitioners and regulatory bodies. The global burden of disease attributable to occupational exposure to lead alone is substantial, contributing to cardiovascular disease, chronic kidney disease and neurological impairment [4]. Current knowledge has firmly established the multi-organ toxicity of these elements. Lead is a potent neurotoxin, mercury targets the central nervous system and kidneys, cadmium is a primary nephrotoxin and carcinogen and arsenic is a well-documented multi-site carcinogen [5, 6]. However, controversies and knowledge gaps persist. The subclinical effects of chronic, low-level exposure are still being elucidated and the synergistic or antagonistic effects of co-exposure to multiple metals are poorly understood [7]. Furthermore, the efficacy and safety of therapeutic interventions like chelation for chronic, low-level exposure remains a subject of debate. This narrative review aims to provide a comprehensive and critical synthesis of the current literature on occupational heavy metal exposure. The objectives are to: (1) review the pathophysiology and major health outcomes associated with exposure to lead mercury, cadmium and arsenic; (2) discuss the current diagnostic and biomonitoring strategies; and (3) critically evaluate the hierarchy of therapeutic and preventive interventions designed to protect worker health. By integrating evidence from toxicological, clinical and epidemiological studies, this review seeks to highlight key challenges and future directions for research and policy in occupational medicine. Therefore, it is of interest to describe the health impacts and preventive strategies related to occupational heavy metal exposure.

## Pathophysiology of heavy metal toxicity:

The mechanisms by which heavy metals exert their toxic effects are multifaceted, but a central unifying pathway is the induction of oxidative stress [8]. Most toxic metals have a high affinity for sulfhydryl (-SH) groups, which are abundant in enzymes and antioxidant molecules. By binding to these groups, metals can inactivate critical enzymes and deplete the cell's primary antioxidant, glutathione (GSH), rendering it vulnerable to damage from reactive oxygen species (ROS) [9]. Beyond this common mechanism, each metal exhibits unique toxicokinetic and toxicodynamic properties.

## Lead (Pb):

Since lead competes with calcium on transporters, lead can also replace calcium, with serious consequences on bone formation, kidney function and, most importantly, neural function (where calcium is indispensable for processes like learning and memory) [10]. By substituting for calcium, lead disrupts intracellular calcium homeostasis and signaling pathways, which are vital for neurotransmitter release, mitochondrial function and apoptosis. This ionic mimicry is a key driver of its profound neurotoxicity [11]. Furthermore, lead interferes with heme synthesis by inhibiting several key enzymes, including aminolevulinic acid dehydratase (ALAD) and ferrochelatase. This disruption leads to the accumulation of precursors like aminolevulinic acid (ALA), which is it-self a neurotoxin and contributes to the microcytic anemia often seen in lead poisoning [12].

## Mercury (Hg):

Elemental and inorganic mercury primarily target the kidneys, while organic mercury (methylmercury) is a potent neurotoxin that can cross the blood-brain barrier [13]. In the kidney, accumulation in the proximal tubules leads to oxidative damage and apoptosis, resulting in tubular dysfunction. Elemental mercury frequently causes damage to the kidneys, central nervous system (CNS), and lungs. Inorganic mercury salts are mostly absorbed through the gastrointestinal tract, with undamaged skin being the secondary route. The salts mostly induce gastrointestinal (GI) and renal damage. Additionally, the GI tract absorbs organic mercury first, followed by undamaged skin. Neurologic signs from organic mercury intoxication are usually delayed [14].

## Cadmium (Cd):

Cadmium is poorly absorbed but has an exceptionally long biological half-life (10-30 years), accumulating primarily in the kidneys and liver [15]. Long-term exposure to cadmium through the air, water, soil, and food causes damage to the skeletal, urinary, reproductive, cardiovascular, central and peripheral neurological, and respiratory systems as well as cancer [16]. Cadmium also disrupts calcium metabolism, contributing to bone demineralization and conditions like Itai-itai disease [17].

## Arsenic (As):

The most prevalent toxic inorganic forms are arsenite (trivalent) and arsenate (pentavalent). In a number of processes, arsenate (pentavalent arsenic) may take the place of phosphate. Phosphate and arsenate share a similar structure and set of characteristics. According to *in vitro* research, arsenate and glucose combine to create glucose-6-arsenate, which is similar to glucose- 6-phosphate and inhibits hexokinase, a crucial enzyme for glycolysis. When compared to arsenate (pentavalent arsenic), arsenite (trivalent arsenic) is thought to be the more hazardous inorganic form. Thiol and sulfhydryl groups, which are important chemical components of many proteins and enzymes found throughout the body, react with arsenite. The proteins and enzymes involved are dysregulated and inhibited as a result of these processes [18].

## Clinical manifestations and health outcomes:

Occupational exposure to heavy metals can result in a wide array of clinical syndromes affecting nearly every organ system. The presentation can be acute, following a high-dose exposure, or chronic and insidious, developing over years of low-level exposure. Chromium, arsenic, cadmium, mercury, and lead are examples of heavy metals (HMs), a class of environmental contaminants that are extremely hazardous and endanger human health. nzyme activity, protein synthesis, and energy metabolism are just a few of the intracellular biochemical processes that HMs disrupt to produce their harmful effects. They can also damage cellular signaling and compromise the integrity of cell membranes, which can result in cellular malfunction and death. HMs has the ability to damage DNA and create gene mutations at the molecular and genetic levels, which can impact genetic expression and transmission [19].

## Neurological effects:

The nervous system is a primary target for several heavy metals. Chronic lead exposure is well-documented to cause both central and peripheral nervous system damage. In adults, this can manifest as cognitive decline, memory loss, mood disorders and a classic peripheral motor neuropathy characterized by wrist drop or foot drop [11]. Acute high-level exposure can lead to a life-threatening encephalopathy with seizures and coma [4]. Mercury is also a potent neurotoxin. Chronic inhalation of elemental mercury vapor in occupational settings (e.g., chlor-alkali plants, dentistry) can cause a classic triad of tremors, gingivitis and a neuropsychiatric syndrome known as erethism (irritability, excitability, memory loss) [20].

## Renal effects:

The type, dosage, method, and length of exposure all affect how much heavy metal harm the kidneys. Nephropathies caused by both acute and chronic intoxication have been shown to range in severity from tubular dysfunctions such acquired Fanconi syndrome to severe renal failure that occasionally results in death [21, 22]. Impaired kidney function, which makes it difficult to properly filter blood, eliminate waste, maintain electrolyte balance, and control blood pressure, is the hallmark of renal dysfunction. Impaired proximal tubular function is the primary characteristic of Pb-induced degenerative alterations in the kidneys, which may develop into Fanconi syndrome. Pb interfered with energy metabolism, calcium homeostasis, glucose regulation, ion transport, and the renin-angiotensin system via interacting with renal cell membranes and enzymes [19].

## Carcinogenic effects:

Several heavy metals are classified as human carcinogens by the International Agency for Research on Cancer (IARC). Arsenic and its inorganic compounds are designated as Group 1 (carcinogenic to humans), causally linked to cancers of the lung, skin and bladder in occupationally exposed populations such as smelter workers and pesticide manufacturers [23, 24]. Cadmium is a known environmental carcinogen that is strongly associated with the development of breast cancer. One common industrial and environmental pollutant that is closely linked to a higher risk of lung cancer is Chromium [19]. The carcinogenic potential of lead is more controversial; IARC classifies inorganic lead compounds as Group 2A (probably carcinogenic to humans), with limited evidence for stomach and lung cancer [25].

## Cardiovascular effects:

There is growing evidence linking chronic heavy metal exposure to cardiovascular disease. Lead is the most studied in this regard, with strong epidemiological evidence showing a dose-dependent association between blood lead levels and an increased risk of hypertension and cardiovascular mortality [26]. The proposed mechanisms include oxidative stress, inflammation and interference with vascular smooth muscle signaling. Chronic arsenic exposure, even at low levels, has been linked to an increased risk of hypertension, atherosclerosis and ischemic heart disease [27].

## Diagnostic approaches and biomonitoring:

Biological monitoring is the cornerstone of assessing exposure, estimating body burden and guiding clinical and public health interventions. The ideal biomarker should be sensitive, specific and reflect the biologically active dose at the target organ. Blood lead level (BLL) is the most widely used biomarker for lead exposure, reflecting recent absorption over the preceding 1-2 months [12]. However, it is a poor indicator of cumulative body burden, as over 90% of lead is stored in bone with a half-life of decades. For research and some clinical contexts, K-shell X-ray fluorescence (KXRF) can be used to non-invasively measure bone lead, providing a better estimate of long-term, cumulative exposure [28]. For mercury, the choice of biomarker depends on the chemical form. Urine mercury concentration is the best indicator of exposure to elemental or inorganic mercury vapor, reflecting accumulation in the kidneys [13]. In contrast, blood mercury levels are more appropriate for assessing recent exposure to organic methylmercury, which is less common in occupational settings outside of specific incidents. Urine cadmium is considered the gold standard biomarker for long-term cadmium exposure and body burden, as it reflects the amount stored in the kidney and correlates well with the risk of renal dysfunction [15]. Cadmium levels can be measured in the blood, urine, hair, nail and saliva samples. A higher arsenic level in a 24-hour urine collection is the most reliable sign of arsenic exposure. Spot urine testing is a possibility in an emergency. Exposure to 50 micrograms/L or more than 100 micrograms of total arsenic is considered confirmed. Urinary arsenic levels after 24 hours usually surpass several thousand milligrams in cases of acute exposure. Usually, spot urine values are higher than 1000 micrograms/L [16, 18].

## Management and preventive interventions:

## Therapeutic management:

The primary and most critical step in managing any case of occupational heavy metal toxicity is the immediate and complete removal of the worker from the source of exposure [29]. The harmful effects of heavy metals have been demonstrated to be minimized by therapeutic approaches like chelation therapy, antioxidants and free radical scavengers, supportive therapy, and exposure reduction and prevention [19]. Chelating agents are compounds that form stable, non-toxic complexes (chelates) with heavy metals, which are then excreted from the body, primarily in the urine. Commonly used agents include calcium disodium ethylenediaminetetraacetic acid (CaNa_2_EDTA) for lead, dimercaprol (BAL) for arsenic and mercury and succimer (DMSA) or unithiol (DMPS) as water-soluble alternatives [30]. In the overall long-term management of lead poisoning, chelation therapy can have short-term benefits; however, these benefits must be accompanied by drastic reduction in environmental exposure to lead if therapy is to have any long-term benefit [31].

## Hierarchy of preventive controls:

Given the limitations of medical treatment, primary prevention is paramount in occupational health. The most effective strategies follow a "hierarchy of controls," which prioritizes interventions from most to least effective [32]:

[1] Elimination/Substitution: The most effective control is to remove the hazard entirely or substitute the toxic metal with a safer alternative. For example, replacing lead-based paints with lead-free alternatives or using lead-free solder.

[2] Engineering controls: If substitution is not feasible, the next step is to isolate workers from the hazard using engineering solutions. This includes process enclosure, local exhaust ventilation (LEV) systems to capture fumes and dust at the source and wet methods to suppress dust generation [33].

[3] Administrative controls: These are changes in work practices and policies to reduce the duration and intensity of exposure. Examples include job rotation, prohibiting eating and drinking in work areas, providing shower and changing facilities and comprehensive worker training on hazards and safe work practices [34].

[4] Personal Protective Equipment (PPE): PPE, such as respirators, gloves and protective clothing, is the last line of defense. While essential, it is the least effective control measure because its efficacy depends on proper selection, fit, maintenance and consistent use by the worker. Over-reliance on PPE is a common failure in occupational safety programs [35].

A comprehensive occupational health program integrates these controls with a robust health surveillance program, including regular workplace air monitoring and worker biomonitoring to ensure that exposure limits are not exceeded and to detect early signs of toxicity.

## Discussion:

This review synthesizes the extensive body of evidence demonstrating that occupational exposure to heavy metals remains a significant global health threat. The pathophysiology, driven largely by oxidative stress and enzymatic inhibition, results in a broad spectrum of diseases affecting multiple organ systems. While acute, high-level poisonings are now less common in high-income countries due to regulation, chronic, low-level exposure continues to be a major concern, contributing to a substantial burden of chronic disease such as hypertension, renal failure and neurocognitive decline [4, 26]. A major gap in the current literature is the limited understanding of the health effects of mixed-metal exposures, which is the reality in many occupational settings like e-waste recycling and smelting [7]. The interactions between metals can be synergistic, antagonistic, or additive, making it difficult to predict health outcomes or establish safe exposure limits based on single-metal studies. Future toxicological and epidemiological research must adopt more complex models to address this reality. Another critical challenge lies in the realm of biomonitoring. While biomarkers of exposure like BLL are well-established, there is a pressing need for more sensitive and specific biomarkers of early biological effect-indicators that can detect subclinical organ damage before irreversible disease develops [36]. For instance, novel urinary biomarkers of kidney injury (e.g., KIM-1, NGAL) may offer an earlier warning of cadmium-induced nephrotoxicity than traditional markers like β2-microglobulin [37]. From a clinical and policy perspective, the findings of this review underscore the inadequacy of a purely reactive approach focused on diagnosis and treatment. Chelation therapy, while life-saving in acute poisoning, has a very limited role in the management of chronic occupational exposure [31]. This highlights the critical need for stronger global standards, technology transfer for engineering controls and robust enforcement mechanisms. The clinical implications are clear: clinicians must maintain a high index of suspicion for heavy metal toxicity in patients with unexplained neurological, renal, or constitutional symptoms, particularly if they have a relevant occupational history. A thorough occupational history is an indispensable diagnostic tool. For public health, the implication is that reducing permissible exposure limits (PELs), as has been done for lead, is a necessary step but is insufficient without a concurrent focus on implementing higher-order engineering and administrative controls to achieve those limits in practice [33].

## Conclusion:

Occupational exposure to heavy metals such as lead, mercury, cadmium and arsenic constitutes a persistent and preventable cause of morbidity and mortality among workers worldwide. The mechanisms are complex, leading to a cascade of cellular damage that manifests as a wide range of neurological, renal, cardiovascular and carcinogenic diseases. While advances in biomonitoring have improved our ability to assess exposure, significant challenges remain in understanding the long-term effects of low-level and mixed-metal exposures and in identifying early markers of disease.

## References

[1] Ahmadi Jalaldehi P (2025). Sci Rep..

[2] Rafiee A (2022). Environ Res..

[3] Zhu Y (2025). Front Public Health..

[4] Papatsoris A (2025). Arch Ital Urol Androl..

[5] Issah I (2021). Rev Environ Health..

[6] Baumert BO (2024). Environ Res..

[7] Dang YM (2025). Free Radic Res..

[8] Bonner EM (2025). J Expo Sci Environ Epidemiol..

[9] Siwakoti RC (2024). Chemosphere..

[10] Radulescu A, Lundgren S (2019). Sci Rep..

[11] Nagaraju R (2022). Crit Rev Toxicol..

[12] Yin T (2025). Ecotoxicol Environ Saf..

[13] Pizarro AB (2022). Cochrane Database Syst Rev..

[14] Posin SL (2023). Mercury Toxicity..

[15] Jiang Z (2025). Environ Res..

[16] Rahimzadeh RM (2017). Caspian J Intern Med..

[17] Nagaraju R (2022). Arch Toxicol..

[18] Kuivenhoven M, Mason K (2023). Arsenic Toxicity..

[19] Cheng Y-F (2025). MedComm..

[20] Vahabi A (2025). Clin Orthop Relat Res..

[21] Barbier O (2005). Nephron Physiol..

[22] García-Muñoz J (2025). Ecotoxicology..

[23] Pillay J (2024). Syst Rev..

[24] National Toxicology Program. (2021). Rep Carcinog..

[25] Sbidian E (2021). Cochrane Database Syst Rev..

[26] Sharma V (2024). Cochrane Database Syst Rev..

[27] Paz-Sabillón M (2023). Biol Trace Elem Res..

[28] Thomas KH (2021). Health Technol Assess..

[29] Spierenburg W (2024). Clin Orthop Relat Res..

[30] Chen J (2025). Ecotoxicol Environ Saf..

[31] Giulioni C (2025). J Basic Clin Physiol Pharmacol..

[32] Janjua S (2021). Cochrane Database Syst Rev..

[33] Moftian N (2025). Sci Rep..

[34] Tattan-Birch H (2022). Cochrane Database Syst Rev..

[35] Contreras EM (2025). PLoS One..

[36] Lipworth L (2025). J Occup Environ Hyg..

[37] Nice KA (2025). Lancet Planet Health..

